# Unique Considerations for Complete Surgical Analgesia for Below-the-Knee Amputations

**DOI:** 10.7759/cureus.14224

**Published:** 2021-03-31

**Authors:** He Li, David H Cho, Vadim Tokhner

**Affiliations:** 1 Department of Anesthesiology, Harbor-University of California, Los Angeles Medical Center, Torrance, USA

**Keywords:** regional anesthesia, posterior femoral cutaneous nerve, below-the-knee amputation

## Abstract

Recent cadaver studies have suggested that posterior femoral cutaneous nerve (PFCN) may contribute to the sensory innervation of the posterior lower leg. Whether this is clinically relevant may be revealed in patients who underwent below-the-knee amputation (BKA) with monitored anesthesia care (MAC) and peripheral nerve blocks. We performed femoral and sciatic nerve blocks for a 55-year-old male patient who underwent BKA and subsequent formalization surgeries as the main surgical analgesia while providing MAC in the operating room. In both cases, the patient could not tolerate surgical incisions in the posteromedial aspect of the lower leg, despite reporting no pain in other areas of the lower leg with surgical stimulation. There may exist a small population of patients in which PFCN makes significant contribution to the sensory innervation of the posterior lower leg. For these patients, the combination of femoral and sciatic nerve blocks may not be adequate in providing surgical analgesia for BKA and related procedures.

## Introduction

Lower extremity amputation is one of the most common vascular surgeries performed in the United States [1]. It is indicated in patients with extensive tissue loss due to trauma or infection, and in patients with peripheral vascular disease who are not candidates for revascularization efforts or failed attempts at revascularization. Regional anesthesia technique is frequently used for lower extremity amputation as it has been shown to be associated with reduced postprocedural pain and short-term need for opiate medications, shorter postanesthesia care unit (PACU) stay, and decreased postoperative cardiopulmonary complications when compared with general anesthesia [2-4]. The femoral and sciatic nerves, as well as their distal branches, are the main block targets for surgical procedures involving the lower leg. The posterior femoral cutaneous nerve (PFCN), which arises from S1-S3 of the sacral plexus and passes through the gluteal region alongside the sciatic nerve, has been recognized conventionally as a nerve that provides sensory innervation to the perineum and posterior thigh to the level of the popliteal fossa. It is not commonly considered as a block target for surgical procedures below the knee. However, there is emerging anatomical evidence suggesting that PFCN may contribute to the sensory innervation of the posterior lower leg [5]. In this report, we present a case in which a combination of femoral and sciatic nerve blocks failed to provide adequate surgical analgesia in the posterior lower leg region for a patient who underwent below-the-knee amputation (BKA) and subsequent formalization surgery. This case may serve as a springboard to investigate the need of performing a PFCN block to improve the analgesia coverage and prevent inadequate blocks for BKA surgeries when inadequate analgesia is initially noted.

## Case presentation

A 55-year-old male with a past medical history of peripheral arterial disease, hypertension, hyperlipidemia, type II diabetes mellitus, end-stage renal disease, and myocardial infarction presented to Harbor-University of California, Los Angeles Medical Center with right foot ischemic necrosis in the setting of local infection. His foot was deemed unsalvageable with vascular bypass surgery, and the decision was made to proceed with guillotine BKA. The anesthetic plan was to perform preoperative femoral and sciatic nerve blocks for surgical analgesia and monitored anesthesia care (MAC) in the operating room. For the sciatic nerve block, the patient was placed in the lateral position and the sciatic nerve was identified under ultrasound with a 6-13 MHz linear probe (S-Nerve, Sonosite, Bothell, WA, USA) as it divides into common peroneal and tibial nerves approximately 5 cm proximal from the popliteal fossa. Then, 30 mL of 0.5% ropivacaine was deposited with an in-plane technique using a 21-G echogenic needle (EchoStim, Havels, Cincinnati, OH, USA) within the common sciatic sheath. Encapsulation of both the tibial and common peroneal nerves was clearly visualized upon injection of the local anesthetics. A femoral nerve block was performed subsequently with the patient in the supine position. The femoral nerve was identified lateral to the femoral artery using ultrasonography, and the needle was inserted laterally to the nerve using an in-plane technique. Subsequently, 20 mL of 0.5% ropivacaine was injected within the femoral sheath. The femoral nerve was seen to be displaced from the fascia iliaca layer above and was appropriately encapsulated by the local anesthetic. After the nerve blocks, the patient reported no pain in the right foot. The patient was then taken to the operating room approximately 30 minutes after the nerve blocks were performed, and while in the operating room he received 2 mg of midazolam and 100 mcg of fentanyl prior to the surgical incision. He reported no pain at the beginning of the surgery until the surgical incision reached to the posteromedial aspect of the lower leg approximately 5-10 cm proximal to the calcaneus. The patient was unable to tolerate the surgery further and the decision was made to convert to general anesthesia with endotracheal intubation. Surgery was completed without incident and no pain medication was given for the reminder of the surgery. Postoperatively, the patient reported no pain in the PACU and did not request any pain medication until 12 hours after the surgery.

Two days after the BKA surgery, the patient was taken back to the operating room for BKA formalization. The anesthetic plan was again to perform peripheral nerve blocks with the addition of an adductor canal catheter for postoperative pain control and MAC in the operating room. The sciatic nerve block was performed in a similar manner as described above. The femoral nerve block was also performed in a similar manner but with the addition of a nerve stimulator to ensure appropriate blockade. An adequate patellar twitch was elicited prior to depositing the local anesthetic medication. The injection was also visualized using ultrasonography. A saphenous nerve catheter at the adductor canal was chosen to minimize postoperative motor blockade. This was performed in the upper third of the thigh with appropriate visualization and spread of the local anesthetics around the saphenous nerve and superficial femoral artery. An indwelling catheter (Stimuplex DIG RC, B Braun Medical, Sheffield, UK) was used and subsequently connected to a continuous infusion pump (On-Q, Avanos, Irvine, CA, USA). Overall, 25 mL, 20 mL, and 10 mL of 0.5% ropivacaine was injected for the sciatic nerve, femoral nerve, and saphenous nerve blocks, respectively. The patient reported 10 out of 10 pain and 3 out of 10 pain before and after the blocks, respectively. In the operating room, the patient reported intolerable pain in the posterior flap region upon testing by the surgical and anesthesia teams, and the decision was made to carry out the surgery under general anesthesia with endotracheal intubation. The patient received a total of 100 mcg of fentanyl for pain intraoperatively. The On-Q pump infusion of 0.2% ropivacaine was started in the PACU at the rate of 5 mL/min. The patient reported no pain upon discharge from the PACU to the inpatient floor. The On-Q pump catheter was discontinued five days later when the patient was discharged to a physical rehabilitation facility.

## Discussion

Sensory innervation of the lower leg is generally attributed to the sciatic and the saphenous nerves. Ultrasound-guided blocks of these two nerves has been shown to be safe and effective in providing surgical analgesia for a wide range of lower leg surgical procedures [6]. At our institution, we commonly perform a combination of femoral nerve block at the level of the femoral crease with sciatic nerve block at the level of popliteal fossa for BKA surgeries carried out under MAC. In the case presented here, the patient experienced significant pain in the posterior lower leg upon surgical stimulation after what appeared to be successful nerve blocks by experienced anesthesia providers. The posterior lower leg in the proximity of the ankle is traditionally considered to be innervated by the posterior tibial nerve, with contribution from the saphenous nerve to the medial side. Feigl et al. recently suggested that PFCN may play an important role in posterior lower leg sensory innervation. In their studies of 83 human cadavers, popliteal ending of the PFCN was found in only 9.7% of the cases, whereas in 44.6% of the cases it was found to end within 10 cm of the most distal point of medial malleolus. In 13.2% of the cases, PFCN showed a close connection to the Achilles tendon [5]. The results of these cadaveric anatomical studies cannot be directly translated into clinical significance, given the fact that the vast majority of the patients tolerate BKA surgeries very well after receiving just saphenous and sciatic nerve blocks [6]. There still may exist a small population of patients in which PFCN plays a much more significant role in posterior lower leg sensory innervation, which may be highlighted by the case for the patient we presented here. Ultrasound-guided PFCN blocks have been used successfully in providing pain control for surgical procedures involving the posterior thigh and in treating neuropathic pain within the inferior gluteal region [7,8]. PFCN blocks aiming at providing perioperative pain relief for lower leg surgical procedures remain an area to be investigated further.

## Conclusions

Future clinical studies designed to address questions such as whether a PFCN block results in improved intraoperative pain control and its impact on postoperative pain score as well as how to identify patients who may benefit from a PFCN block in addition to the conventional nerve blocks will be helpful. Clinical considerations should also be made as to whether the PFCN can be effectively blocked along with the sciatic nerve in a single injection. Such future work may shed new light on the clinical relevance of PFCN in lower leg sensory innervation.
